# A novel uromodulin mutation in autosomal dominant tubulointerstitial kidney disease: a pedigree-based study and literature review

**DOI:** 10.1080/0886022X.2018.1450757

**Published:** 2018-03-23

**Authors:** Ziqiang Lin, Juan Yang, Hong Liu, Dan Cai, Zhenmei An, Yerong Yu, Tao Chen

**Affiliations:** a Department of Endocrinology and Metabolism, West China Hospital of Sichuan University, Chengdu, P. R. China;; b Department of Endocrinology, Guihang 302 Hospital, Anshun, P. R. China;; c Department of Endocrinology, Science City Hospital of Sichuan Province, Mianyang, P. R. China;; d Department of Endocrinology, PI County People’s Hospital, Chengdu, P. R. China

**Keywords:** Autosomal dominant tubulointerstitial kidney disease, familial juvenile hyperuricemic nephropathy type 1, uromodulin gene mutation, juvenile gout, hyperuricemia

## Abstract

Autosomal dominant tubulointerstitial kidney disease caused by mutations in uromodulin gene (ADTKD-*UMOD*) is a spectrum of hereditary renal disorders, characterized by early-onset hyperuricemia, gout and progressive nephropathy. This study presented a novel *UMOD* mutation in an ADTKD pedigree and reviewed studies in Chinese population. The index patient is a 16-year-old girl with hypertension, hyperuricemia and normal serum creatinine level. Four affected and six unaffected members were available for genetic screen. The mutation analysis was performed by next-generation sequencing and direct sequencing. A literature research was conducted to review Chinese ADTKD-*UMOD* cases. MEDLINE and Chinese Biomedicine Databases were searched with ‘uromodulin’, ‘juvenile gout’ and their related terms. Genetic sequencing revealed a *de novo* mutation within exon 3 (Cys223Gly), which was co-segregating with phenotype in this pedigree. In the review, four studies and our study involving a total of 67 ADTKD patients from 11 families were identified. Of these patients, 27 were confirmed to carry *UMOD* mutations. Mutations occurred in exon 3 were commonly observed, while mutations within exon 4, 5 and 9 occurred less frequently in Chinese ADTKD-*UMOD* cases. Among these cases, median age of symptom onset was 26.5 years, median age of end-stage renal diseases (ESRD) or death by ESRD was 41.9 years without renal replacement treatment. Phenotype caused by mutations in D8C domain seemed to be severe than those in GPI domain. Compared with patients of other race, Chinese ADTKD-UMOD patients advanced more aggressively to ESRD.

## Background

Autosomal dominant tubulointerstitial kidney disease caused by UMOD pathogenic variants (ADTKD-UMOD) was previously known as familial juvenile hyperuricemic nephropathy type 1 (FJHN1), medullary cystic kidney disease type 2 (MCKD2) and *UMOD*-associated kidney disease [1,2]. This disease was characterized by early-onset hyperuricemia, gout and hypertension, reduced fractional renal urate excretion and progressive interstitial nephropathy [3]. Mean age of progression to end-stage renal disease (ESRD) was 56 years old [4]. Heterogeneous mutations in the *UMOD* gene located on chromosome 16p12.3–p13.11 [5,6].

Uromodulin, encoded by *UMOD* gene, is a 640-amino-acid glycoprotein extensively detected in human urine [7,8]. Uromodulin is synthesized in thick ascending limb and early distal convoluted tubule of kidney, expressed on luminal membrane and released into urine through proteolytic cleavage by hepsin [9–11]. Uromodulin contains in the N-terminal region three epidermal growth factor (EGF)-like domains, an eight cysteine domain (D8C), a zona pellucid (ZP) domain and a glycosylphosphatidylinositol (GPI) anchor segment in the C-terminal [12]. Mutations in *UMOD* coding regions, such as exon 3, 4, 5, 6 and 9, could lead to a delay in maturation rate of the protein [4,13–15]. Immature uromodulin was trapped in the endoplasmic reticulum (ER), and subsequently expressed or released by the cellular membrane in a less efficient manner [16,17]. So far, more than 60 *UMOD* mutations have been identified to contribute to ADTKD pathogenesis [3,18–23]. Most are missense mutations and small in-frame deletions within exon 3 and exon 4, where the EGF-like domains and D8C are encoded [18–20].

ADTKD-*UMOD* cases have been widely identified in western countries and some regions of Asia including Japan, South Korea and India [4,24–26]. However, there has been very few report from China. Hence, this study reported a novel *UMOD* mutation in a Chinese family, and comprehensively reviewed studies regarding ADTKD-*UMOD* cases of Chinese ethnic, in order to provide further evidence for the underlying mechanism of this rare disease.

## Materials and methods

### Patients

**Figure 1.**
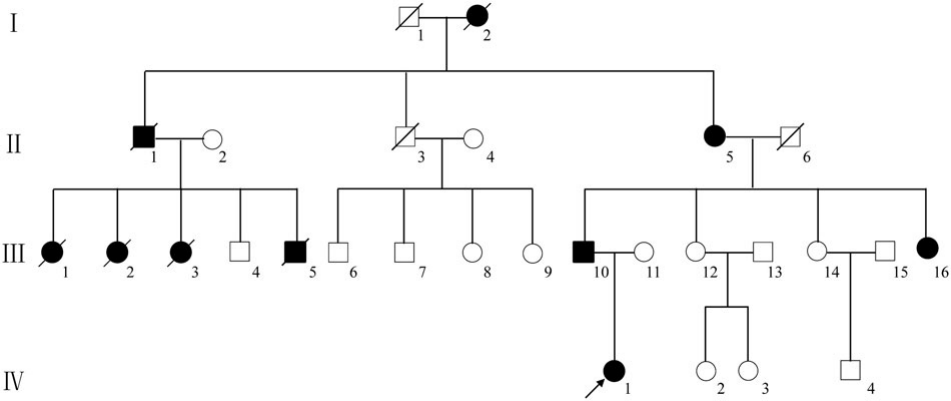
Pedigree of the ADTKD-*UMOD* family.

The index patient was a 16-year-old female admitted to the Department of Endocrinology and Metabolism at West China Hospital of Sichuan University in January 2016 with hypertension (160/90 mmHg) and elevated uric acid level (>7 mg/ml). The patient had a positive family history of early-onset gout, hyperuricemia and ESRD. The clinical information of the patient’s parents and one paternal aunt was collected during interview, and those of other family members (Figure 1), including gender, age, symptoms, positive test results, age at symptom onset and treatments, were documented according to narrative of two members. Blood samples from the index patient and her parents and nail samples from the other six family members (one patient and five unaffected individuals) were collected for genetic screen.

### Genetic sequencing

Genomic DNA was extracted from blood and nail tissues using TianGen DNA Extraction Kit. Next generation sequencing was applied to capture suspicious mutation sites of the index patient (Illumina HiSeq-2500 system), then the suspected mutation was confirmed by direct sequencing to the index patient and other available individuals (Joy Orient Translational Medicine Research Center Co., Ltd).

### Search strategy

To comprehensively review ADTKD-*UMOD* cases in the Chinese population, we searched for primary studies published by October 2017 on MEDLINE (PubMed) and Chinese Biomedical Database. Mesh term ‘juvenile gout’ and its related terms including ‘Familial juvenile hyperuricemic nephropathy’, and ‘uromodulin-associated kidney diseases’ were applied for literature search. Articles written in English and Chinese languages were independently screened by two authors (J Yang and T Chen). All published full-text case studies on Chinese ADTKD-*UMOD* families were included. Available clinical data, including number of affected members, type of *UMOD* mutation, initial manifestation, age at symptom onset, ESRD status and age of death were sorted out and analyzed.

## Results

### Clinical features of a ADTKD-UMOD family

#### Proband

The index patient (IV-1) was a 16-year-old girl who complained of recurrent headache for 2 years. At the age of 14, she was found to have increased hyperuricemia with hypertension (160/90 mmHg). Laboratory examinations on admission revealed slightly impaired renal function (eGFR 78.64 mL/min/1.73 m^2^, serum creatinine 93 μmol/L and serum urea 10.4 mmol/L), increased uric acid level (8 mg/dl) and decreased fraction excretion of uric acid (5.73%). Ultrasonography showed slightly enhanced echo in bilateral kidneys, urine acid crystals in the right kidney and stones in the left kidney. Subsequent laboratory workup ruled out other causes of secondary hypertension involving renal artery disease and endocrine hypertension. The patient refused to undergo renal biopsy.

#### Other affected family members

There were nine other affected individuals in this family, who developed chronic kidney diseases (CKD) of an autosomal dominant trait (Figure 1). The clinical features of these patients were described in Table 1. Seven of them suffered from hyperuricemia and gout before the age of 40, five died from end-stage renal diseases (ESRD) between 36 and 45, only three stayed alive. Among alive individuals, one 63-year-old female patient (II-5) developed gout and hyperuricemia at the age of 39, progressed to ESRD at 61 and started hemodialysis since then. After hemodialysis, her serum uric acid level and blood pressure returned to normal, acute flare ceased. One 43-year-old male patient (III-10) developed gout at the age of 12, CKD at 21, hypertension at 31, advanced to ESRD at 40, and he underwent peritoneal dialysis at 41 and received renal transplantation at 42. Afterwards, his serum uric acid and serum creatinine levels returned to normal, and his blood pressure was well controlled by irbesartan. The other 32-year-old female patient (III-16) was diagnosed with hyperuricemia at the age of 32, but she refused to take any medicine due to plan for pregnancy. Her serum creatinine slightly increased to 113 μmol/L and eGFR decreased to 65.8 mL/min/1.73m^2^, while her blood pressure was within normal range and she had not developed gout at this stage.

**Table 1.** Summary of *UMOD* mutations and clinical features in Chinese affected families.

Author/Year	*n*/*N*	Exon	Domain	Mutations	Age of onset^a^	Initial manifestation of index patients	Age at ERSD or death^a^
Wei/2012 [19]	7/13	9	GPI	c.1815A/G, p.Thr605Gly	12–45	HUA, HTN, CKD	46–51
Liu/2013 [21]	3/3^b^	3	EGF3	c.326T/A, p.Val109Glu	18	HUA, HTN, CKD	21
	3/6	3	D8C	c.707C/A; p.Pro236Gln	24–35	HUA	35–41
	3/4	3	D8C	c.744C/G, p.Cys248Trp	11–18	HUA, CKD, HTN	21–41
Huang/2015 [27]	2/12	4	D8C	c.854C/A, p.Ala285Glu	27	Gout, HUA	40–47
Xia/2015 [28]	1/4	3	EGF1	c.197T/C, p.Leu66Pro	NA	NA	NA
	1/8	3	D8C	c.272delC	NA	NA	NA
	1/NA	3	D8C	c.744C/G, p.Csy248Trp	NA	NA	NA
	1/NA	3	D8C	c.707C/G, p.Pro236Gln	NA	NA	NA
	1/2	5	ZP	c.1153C/T, p.Arg385Trp	NA	NA	NA
This study	4/9	3	D8C	c.667T/G, p.Cys223Gly	12–32	HUA, Gout, HTN	36–61

CKD: chronic kidney disease; D8C: domain of eight cysteines; ZP: D8C: domain of eight cysteines; EGF: epidermal growth factor like domain; ESRD: end-stage renal disease; GPI: glycosylphosphatidylinositol segment; HTN: hypertension; HUA: hyperuricemia; *n*/*N*: affected family members/number of screened patients/; NA: not available.

a Data from part of patients with available information.

b Two mutation carriers remained asymptomatic.

### Mutation in the UMOD gene

**Figure 2.**
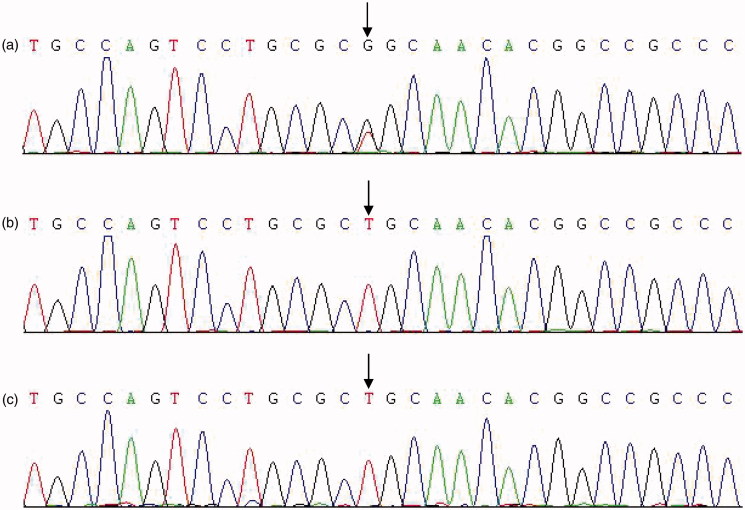
Mutation in *UMOD* gene. (a) Sequence of an affected individual; the site of the missense mutation p.Cys223Gly (c.667T✓G) is shown with an arrow. (b) Sequence of an unaffected individual. (c) Sequence of a healthy normal control.

Next-generation sequencing identified a potential mutation in exon 3 of *UMOD* gene in the index patient. The novel heterozygous missense mutation (c0.667 T✓G, p.223, Cysteine✓Glycine) was further confirmed by direct sequencing in the index patient and the other three tested patients. Whereas, six unaffected family members carried wild-type *UMOD* gene (Figure 2). The autosomal dominant mutation manifested co-segregation of disease phenotypes in this affected family (Figure 1). We searched Exac database using term ‘UMOD’, and retrieved no report on Cys233Gly in Chinese individuals and other race.

### Review of ADTKD-UMOD in Chinese

A comprehensive review on ADTKD-*UMOD* cases in the Chinese population was implemented. Data from four primary studies involving Chinese ADTKD-*UMOD* cases [19,21,27,28] along with the present study were incorporated (Tables 1 and 2). Nine *UMOD* mutations were discovered in 11 Chinese families involving 67 affected patients, 27 of whom were genetically confirmed. Hyperuricemia was the first presented manifestation in most cases (77.1%), at median age of 26.5 years (*n* = 22). Gout commonly occurred in male and in female with advanced stage of CKD. Hypertension usually developed after renal failure. While, the index patient (16-year-old) in this study and another case (21-year-old) presented hypertension and hyperuricemia with normal serum creatinine level. Median age at ESRD development or death were 41.9 years (range from 21 to 61, *n* = 20). Patients with mutation in D8C domain developed HUA and ESRD at younger ages compared with those in GPI domain (20.5/40.5 vs. 26.0/48.0 years, *p* = .42, *p* = .006, respectively). Through the treatment of dialysis and kidney transplantation, two patients were reported to be relieved from symptoms of hyperuricemia and gout, and had blood pressure under control.

**Table 2.** The age of onset of hyperuricemia and ESRD in different affected UMOD domain.

UMOD domains	Median age at HUA	Median age at ESRD
D8C	20.5 (12.5,30.5), *n* = 12	40.5 (38.3, 44.3)^a^, *n* = 14
GPI	26 (16, 32), *n* = 7	48.0 (47.0, 50.5), *n* = 5
EGF3	18, 20^b^, *n* = 2	21^b^
ZP	24, *n* = 1	–

a *p* = .006 compared whit mutations in GPI

b Only two case of mutation in EGF3 domain in the present study, only 1 of them advanced to ESRD.

## Discussion

ADTKD-*UMOD* is defined by the presence of a heterozygous pathogenic variant in *UMOD* gene, encoding uromodulin, in hereditary tubulointerstitial kidney diseases. Most patients with ADTKD-*UMOD* have elevated serum creatinine and serum uric acid level as well as reduced fractional excretion of uric acid [17,29]. It is a very rare autosomal dominant disease that the true incidence of it has remained unclear. Nearly 70 mutations leading to ADTKD-*UMOD* have been discovered worldwide, most frequently in the United States [30,31] and European countries [4,18,32]. Only eight mutations from ten affected families were recently identified in China [19,21,27,28]. This study reported a family with a novel *UMOD* mutation, Cys223Gly, and as the first one reviewed the currently reported ADTKD-*UMOD* cases of Chinese ethnic.

As like the case in other ethnics [14,15,24,31], *UMOD* mutations in the Chinese population are mostly located within exon 3 and 4, which account for 61–93% of the reported mutations. These regions encode the three EGF-like domains as well as the D8C of uromodulin. Of the mutations in exon 3, two mutations (Val109Glu and Leu66Pro) and two others (Pro236Gln and Cys248Tyr) wreaked missense mutations located in EGF-like domains and D8C, respectively; another one, p. Cys272 del, led to deletion mutation in D8C. The other three mutations, Ala285Glu, Arg385Trp and Thr605Gly occurred in exon 4, 5 and 9, respectively. Demolition of EGF-like, D8C, ZP and GPI-anchor segments lead to disrupted trafficking of uromodulin, endoplasmic reticulum storage of protein, altered formation of apical plasma membrane, and finally decreased uromodulin expression and excretion [12,17]. In this study, we identified a novel mutation Cys223Gly in exon 3. This mutation caused the amino acid replacement of cysteine with glycine at position 223 due to a base mispairing (c0.667 T/G) mapping to D8C. Similar to another variant (c0.667 T/C, p.Csy223Arg) at this site, replacement of cysteine may hinder the maturation of precursor uromodulin and subsequently led to abnormal protein construction [12].

Moskowitz et al. comprehensively reviewed the phenotype of 202 patients Uromodulin-associated kidney disease from 74 families, most of them were Caucasian [4]. The results showed that median ages at onset of hyperuricemia and ESRD were 24 and 56 years, respectively [4]. Compared with these patients, Chinese patients seemed to have equivalent onset-age of hyperuricemia (26.5 years), but progressed in a more aggressive manner to ESRD (41.9 years), about 14 years earlier than the former [4]. Interestingly, the earlier onset of ESRD among patients with mutations in the D8C domain compared with those in the other domain consisted with the finding in the previous study [4]. Other risk factors concerning high susceptibility, late diagnosis and undertreatment in undeveloped regions may also contribute to the poor prognosis of Chinese patients.

One limitation of our study revealing the novel *UMOD* mutation is that the affected patients refused to undertake renal biopsy, which made it difficult to further investigate tubulointerstitial nephropathy at histological and protein levels. Despite of the shortage of publications regarding this topic, the clinical characteristics summarized in this study may have reflected an overall picture of Chinese ADTKD-*UMOD* based on currently available data. By summarizing the clinical cases and identifying relevant mutations in *UMOD* gene, more evidences were presented to better understand the pathologic condition and underlying mechanism of ADTKD-*UMOD* in the Chinese population.

## Conclusion

This study identified a novel mutation Cys223Gly in exon 3 of *UMOD* gene which induced ADTKD in a Chinese family. Literature review summarized clinical features of this disease in Chinese population. This study stressed the importance of the *UMOD* gene tests in diagnosis of a spectrum of early-onset hyperuricemia with renal disorders and/or family history of renal disorders.
